# Identifying Patterns of Stigmatizing Language Use in the Safety Net

**DOI:** 10.1007/s11606-026-10592-3

**Published:** 2026-06-24

**Authors:** Kriti Gogia, Zeyu Li, Kara Simpson, Nichola J. Davis, Remle Newton-Dame

**Affiliations:** 1 NYC Health + Hospitals, New York, NY USA; 2 NYU School of Medicine, New York, NY USA

**Keywords:** stigmatizing language, bias, safety net

## INTRODUCTION

Stigmatizing language “transmits” bias between providers, negatively impacts clinical care,^1^ and reduces patient trust.^2^ Identifying and mitigating stigmatizing language is imperative for systems serving low income and historically minoritized communities.^3,4^ NYC Health + Hospitals is the nation’s largest municipal safety net system, serving one million New Yorkers annually. We sought to identify patterns of stigmatizing language use, racial bias and impacted populations, to inform mitigation strategies and delivery of equitable, high-quality care.

## METHODS

We implemented a flexible, rapid lexicon search strategy,^5^ selecting literature-derived stigmatizing terms. To select final terms, we convened an internal group of clinicians, informaticists and equity leaders.^1,3^ Disease-specific terms were excluded. An exact string search identified terms within clinical notes for adults (≥ 18 years) with an inpatient (IP), outpatient (OP) or emergency department (ED) visit in NYC Health + Hospitals from July 2022—June 2023. We conducted 15 manual chart reviews to exclude terms that were hard-coded in EHR templates or diagnoses. No external validation was undertaken. Final terms were non-cooperative, argumentative, combative, belligerent, confrontational, difficult patient, hysterical, unmotivated, and unpleasant (including grammatical permutations).

We measured stigmatizing language prevalence at note level, stratified by provider type and care setting. We compared patient level prevalence across socio-demographics and chronic comorbidity. Significance was assessed via Pearson’s chi squared in R Studio. We modeled the odds of ≥ 1 stigmatizing term in the EMR using a multivariable, logistic Generalized Estimating Equation (GEE), accounting for facility clustering while minimizing Quasi-likelihood under Independence Criterion in SAS Viya. The final patient-level model adjusted for EHR-documented race/ethnicity, age, sex, language, payor, homelessness status, Elixhauser Comorbidity Index, diagnosed alcohol or substance use disorder (SUD/AUD), primary care visits, OP visits and ED visits. BRANY IRB approved this study.

## RESULTS

**Figure 1:**
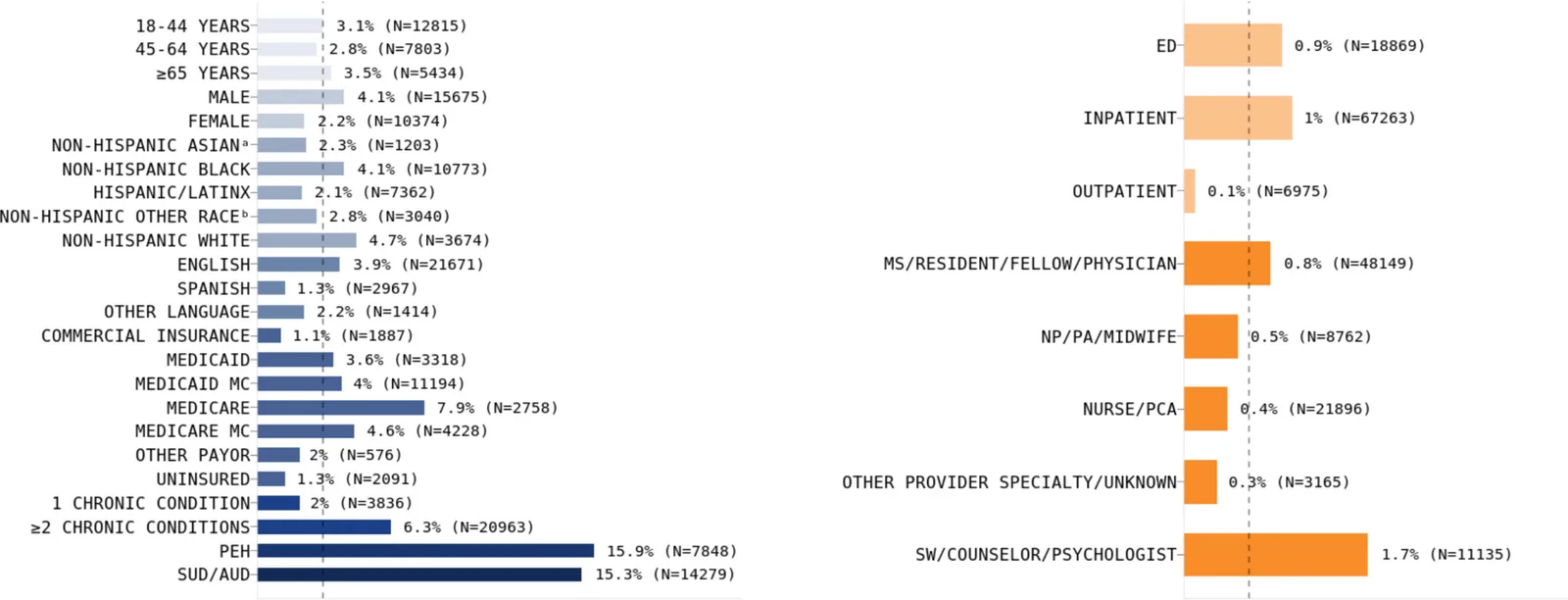
Number (N) and percentage (%) of 1+ stigmatizing terms. On the left, patient-level prevalence by socio-demographics and clinical characteristics. On the right, note-level prevalence by care setting and provider type. Dotted line in both panels represents the overall prevalence, 3.1% for patients and 0.6% for notes. MC: managed care. PEH: patients experiencing homelessness. SUD/AUD: substance/alcohol use disorder. ED: emergency department. MS: Medical Student. NP: Nurse Practitioner. PA: Physician Assistant. PCA: Patient Care Associate. SW: Social Worker. ^a^ includes Native Hawaiian and Pacific Islander. ^b^ includes Native American, Alaskan Native, Two or more races and Unknown.

**Figure 2:**
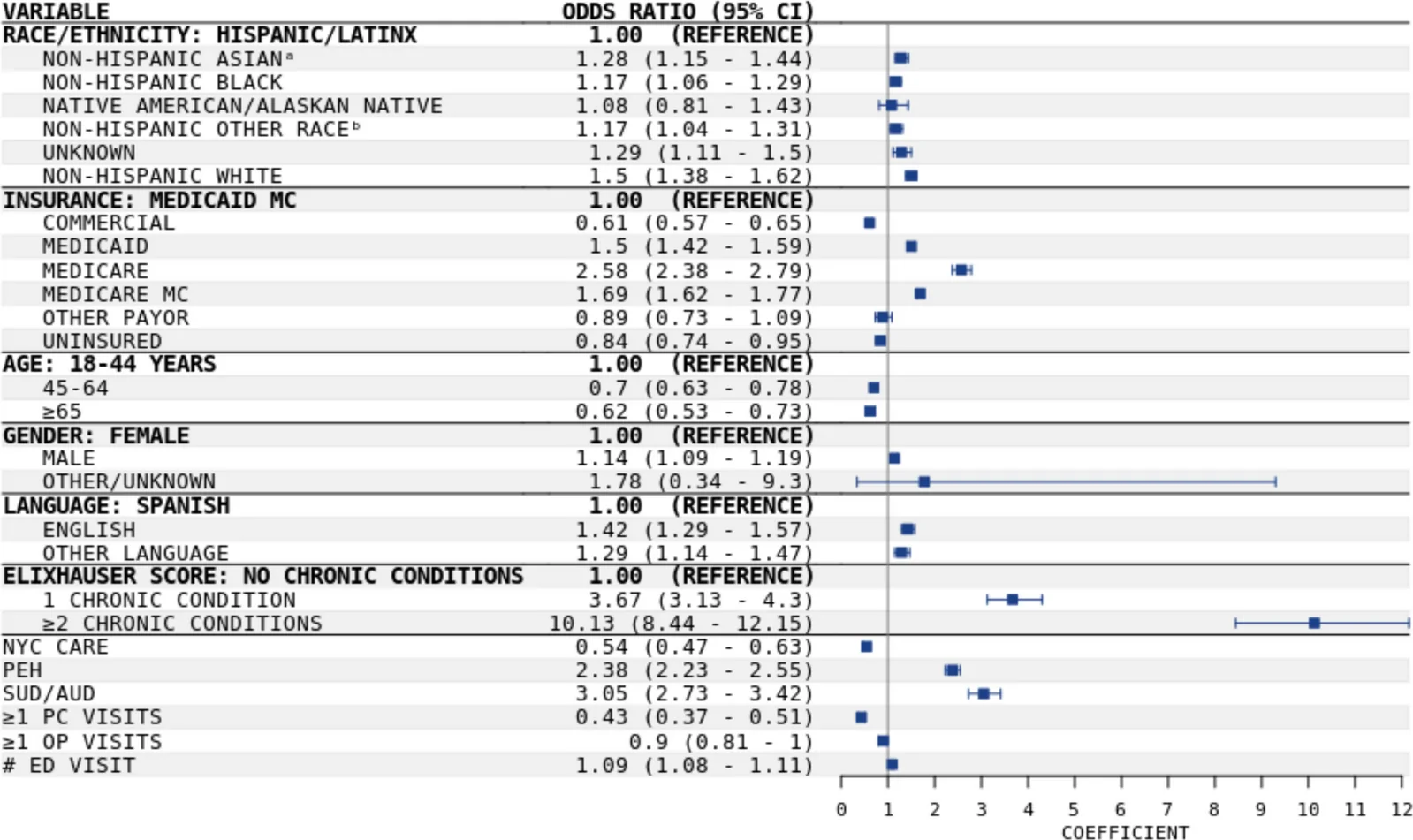
Adjusted odds ratios from the GEE regression model. MC: managed care. NYC care: internal insurance option. PEH: patients experiencing homelessness. SUD/AUD: substance/alcohol use disorder. PC: primary care. OP: outpatient. ED: emergency department. ^a^ includes Native Hawaiian and Pacific Islander. ^b^ includes Two or more races.

From July 2022-June 2023, 851,130 patients were seen, generating 15,006,940 notes. Stigmatizing terms were present in 93,107 medical notes (0.6%, Fig. 1) representing 26,052 unique patients (3.1%). Prevalence was highest amongst notes authored by Social Workers, Counselors or Psychologists (1.7%). Prevalence was < 1% among other doctors, nurses and care team members. Stigmatizing term use was highest in IP (1.0%) and ED (0.9%) notes, compared to OP notes (0.1%). Prevalence was highest among patients with experience of homelessness (15.9%), SUD/AUD (15.3%), Medicare insurance (7.9%) and ≥ 2 chronic comorbidities (6.3%). After adjustment, influential patient-level factors (Fig. 2) were ≥ 2 chronic conditions (OR 10.1), Medicare insurance (2.6), SUD/AUD diagnosis (3.1) and homelessness (2.4).

Significant racial variation was observed, with double crude prevalence for Non-Hispanic White (4.7%) and Black (4.1%) patients compared to Hispanic/Latinx patients (2.1%, *p* < 0.001). After adjustment, odds remained elevated for White (1.5) and Black patients (1.2).

## DISCUSSION

This study identified “hotspots” of stigmatizing language across 93,107 notes and 26,052 patients at the nation’s largest municipal safety net system. Stigmatizing language was concentrated in acute notes, written by Social Workers, Counselors or Psychologists, who predominantly served patients with behavioral health needs. Prevalence was highest among patients with documented multi-morbidity, homelessness and SUD/AUD, replicating findings from the literature.^3,6^ After adjustment, these factors remained influential, underscoring the need for further research into vulnerable populations across all races.

This study likely underestimates true stigmatizing language prevalence within our health system. Our rapid lexicon search using uniformly negative terms was less comprehensive than gold-standard natural language processing^7^ or AI, which can better differentiate context and nuance (e.g. “substance abuse” vs “domestic abuse”). However, identifying broad hot spots and populations most impacted by stigmatizing language allowed us to tailor loci of intervention to maximize impact. The subsequent system improvement efforts include remediating EHR-based language and investing in culture change, with an emphasis on vulnerable populations. A SUD/AUD pilot is replacing stigmatizing language with neutral terms (e.g., from “substance abuse” to “substance misuse”) in frequently used EHR note templates and behavioral health screeners. An ongoing communication and workforce training campaign aims to provide better care for all patients by emphasizing respect and dignity in our words and actions. Using a rapid lexicon search is allowing our systems to monitor progress in stigmatizing term reduction without significant IT and analyst resources.

Addressing stigmatizing language matters. Negative language in patient charts can enhance disparities^1,3^ and worsen treatment outcomes.^7^ A rapid evaluation strategy allows safety-net systems to tailor interventions, improving equitable and dignified care for all patients.

## Data Availability

The data that support the findings of this study contain Protected Health Information and cannot be made publicly available due to patient privacy concerns.
